# Chocolate consumption and all-cause and cause-specific mortality in a US population: a post hoc analysis of the PLCO cancer screening trial

**DOI:** 10.18632/aging.203302

**Published:** 2021-07-29

**Authors:** Guo-Chao Zhong, Tian-Yang Hu, Peng-Fei Yang, Yang Peng, Jing-Jing Wu, Wei-Ping Sun, Long Cheng, Chun-Rui Wang

**Affiliations:** 1 Department of Hepatobiliary Surgery, The Second Affiliated Hospital of Chongqing Medical University, Chongqing, China; 2 Department of Cardiology, The Second Affiliated Hospital of Chongqing Medical University, Chongqing, China; 3 Department of Nephrology, The Second Affiliated Hospital of Chongqing Medical University, Chongqing, China; 4 Department of Geriatrics, The Fifth People’s Hospital of Chengdu, Chengdu, China; 5 Department of Nutrition and Food Hygiene, School of Public Health and Management, Chongqing Medical University, Chongqing, China; 6 Department of Gastrointestinal Surgery, The Second Affiliated Hospital of Chongqing Medical University, Chongqing, China; 7 Department of Breast and Thyroid Surgery, The Second Affiliated Hospital of Chongqing Medical University, Chongqing, China; 8 Department of Infectious Diseases, Institute for Viral Hepatitis, The Key Laboratory of Molecular Biology for Infectious Diseases, Chinese Ministry of Education, The Second Affiliated Hospital of Chongqing Medical University, Chongqing, China

**Keywords:** chocolate, mortality, cardiovascular disease, cancer

## Abstract

Few studies with mixed results have examined the association between chocolate consumption and mortality. We aimed to examine this association in a US population. A population-based cohort of 91891 participants aged 55 to 74 years was identified. Chocolate consumption was assessed via a food frequency questionnaire. Cox regression was used to estimate risk estimates. After an average follow-up of 13.5 years, 19586 all-cause deaths were documented. Compared with no regular chocolate consumption, the maximally adjusted hazard ratios of all-cause mortality were 0.89 [95% confidence interval (CI) 0.84–0.94], 0.84 (95% CI 0.79–0.90), 0.86 (95% CI 0.81–0.93), and 0.87 (95% CI 0.82–0.93) for >0–0.5 servings/week, >0.5–1 serving/week, >1–2 servings/week, and >2 servings/week, respectively (*P*_trend_ = 0.009). A somewhat stronger inverse association was observed for mortality from cardiovascular disease and Alzheimer’s disease. A nonlinear dose–response pattern was found for all-cause and cardiovascular mortality (all *P*_nonlinearity_ < 0.01), with the lowest risk observed at chocolate consumption of 0.7 servings/week and 0.6 servings/week, respectively. The favorable associations with all-cause and cardiovascular mortality were found to be more pronounced in never smokers than in current or former smokers (all *P*_interaction_ < 0.05). In conclusion, chocolate consumption confers reduced risks of mortality from all causes, cardiovascular disease, and Alzheimer’s disease in this US population.

## INTRODUCTION

Because of its exquisite taste and flavor, chocolate is a widely consumed confectionery product, especially in Western countries [1]. In 2018/2019, it is estimated that around 7.7 million tons of chocolate confectionery were consumed globally [2]. Mounting studies have investigated health effects of chocolate [3]. Several meta-analyses found that chocolate consumption was inversely associated with risks of stroke, coronary heart disease, and heart failure [4–6], whereas other studies found that chocolate consumption was not significantly associated with the risk of overall invasive cancer [7] or was positively associated with risks of breast and colorectal cancers [7, 8].

However, evidence on the association of chocolate consumption with mortality is sparse and somewhat mixed. Specifically, higher chocolate consumption was found to be significantly associated with lower mortality from all causes [9] and cardiovascular disease (CVD) [9, 10], whereas no significant association was observed for chocolate consumption and coronary heart disease mortality [11]. These inconclusive results may be due to the differences in study population, study outcome, and/or study method. A study of 470 elderly men observed that higher cocoa intake conferred lower all-cause and cardiovascular mortality [12]. However, findings from this study may not be applicable to chocolate, as cocoa is only a natural product of cocoa beans while chocolate is a solid food product mainly including cocoa, cocoa butter, and sugar [13]; furthermore, this study has a small sample size, and could be subject to small study effects (i.e., small studies are conducted with less methodological rigor and tend to report larger risk estimates compared with large studies) [14].

Currently, evidence on the association between chocolate consumption and health outcomes is weak [15]. Given the need for more data on this topic, we conducted a post hoc analysis of the Prostate, Lung, Colorectal, and Ovarian (PLCO) Cancer Screening Trial to examine the hypothesis that chocolate consumption confers reduced risks of all-cause and cause-specific mortality in a US population.

## RESULTS

### Participant characteristics

In the whole study population, the mean (standard deviation) age was 65.3 (5.7) years; the proportion of males was 46.3% (Table 1); and the average chocolate consumption (standard deviation) was 1.5 (3.2) servings/week. Compared with chocolate non-consumers, those consuming >2 servings/week were less likely to have a history of diabetes or hypertension, had higher energy intake from diet but lower Healthy Eating Index-2015. Moreover, participants consuming >2 vs. 0 servings/week of chocolate had higher consumption of red meat, processed meat, dairy, and coffee but lower consumption of fruit and vegetable, and higher intakes of saturated fatty acids, added sugar, sodium, magnesium, potassium, and calcium.

**Table 1:** Baseline characteristics of study population according to chocolate consumption^a^.

**Characteristics**	**Energy-adjusted chocolate consumption (servings/week)**					***P***
	**0**	**>0–0.5**	**>0.5–1**	**>1–2**	**>2**	
No. of participants	5368	40760	14821	10907	20035	<0.001
Age (years)	65.8 ± 5.7	65.3 ± 5.6	65.3 ± 5.7	65.0 ± 5.7	65.2 ± 5.8	<0.001
Male	2907 (54.2)	18217 (44.7)	7090 (47.8)	5097 (46.7)	9232 (46.1)	<0.001
Ethnic group						
Non-Hispanic white	4372 (81.4)	36249 (88.9)	13702 (92.4)	10236 (93.8)	18989 (94.8)	<0.001
Non-Hispanic black	433 (8.1)	1619 (4.0)	381 (2.6)	209 (1.9)	339 (1.7)	
Hispanic	140 (2.6)	655 (1.6)	208 (1.4)	129 (1.2)	228 (1.1)	
Others^b^	423 (7.9)	2237 (5.5)	530 (3.6)	333 (3.1)	479 (2.4)	
Married or living as married	4000 (74.5)	31975 (78.4)	11695 (78.9)	8695 (79.7)	15568 (77.7)	<0.001
Body mass index (kg/m^2^)	27.1 ± 5.1	27.0 ± 4.8	27.4 ± 4.8	27.2 ± 4.7	27.3 ± 4.8	<0.001
Educational degree						
College below	3512 (65.4)	25735 (63.1)	9479 (64.0)	6790 (62.3)	12649 (63.1)	<0.001
College graduate	884 (16.5)	7227 (17.7)	2652 (17.9)	1948 (17.9)	3621 (18.1)	
Postgraduate	972 (18.1)	7798 (19.1)	2690 (18.1)	2169 (19.9)	3765 (18.8)	
Alcohol consumption (g/day)	10.6 ± 29.6	10.3 ± 26.6	9.8 ± 26.5	8.7 ± 21.5	8.1 ± 21.6	<0.001
Smoking status						
Current	442 (8.2)	3472 (8.5)	1383 (9.3)	982 (9.0)	2199 (11.0)	<0.001
Former	2418 (45.0)	17274 (42.4)	6261 (42.2)	4417 (40.5)	7983 (39.8)	
Never	2508 (46.7)	20014 (49.1)	7177 (48.4)	5508 (50.5)	9853 (49.2)	
Physical activity (min/week)^c^	130.2 ± 133.9	124.9 ± 123.8	122.2 ± 121.3	121.0 ± 120.8	117.0 ± 120.5	<0.001
Energy intake from diet (kcal/day)	1641.8 ± 747.4	1567.7 ± 658.4	1748.0 ± 716.5	1840.3 ± 729.8	2040.6 ± 789.7	<0.001
Healthy Eating Index-2015	68.8 ± 9.9	68.2 ± 9.5	66.5 ± 9.4	65.8 ± 9.3	63.1 ± 9.5	<0.001
History of diabetes	908 (16.9)	2651 (6.5)	643 (4.3)	397 (3.6)	687 (3.4)	<0.001
History of hypertension	1810 (33.7)	12628 (31.0)	4502 (30.4)	3115 (28.6)	5860 (29.2)	<0.001
Family history of cancer	2828 (52.9)	22711 (55.9)	8410 (56.9)	6137 (56.4)	11439 (57.2)	<0.001
Aspirin user	2274 (42.6)	17605 (43.4)	6494 (44.0)	4842 (44.6)	8917 (44.7)	0.004
Food consumption						
Red meat (g/day)	54.1 ± 54.9	53.2 ± 46.1	64.9 ± 51.7	69.0 ± 55.6	72.7 ± 58.4	<0.001
Processed meat (g/day)	16.8 ± 23.1	14.8 ± 16.9	17.7 ± 18.7	18.4 ± 19.0	19.4 ± 19.9	<0.001
Fruit (g/day)	312.2 ± 276.0	272.7 ± 219.7	266.8 ± 200.5	273.0 ± 204.1	270.5 ± 209.5	<0.001
Vegetable (g/day)	319.1 ± 238.2	278.3 ± 187.1	280.3 ± 178.1	285.3 ± 173.7	285.4 ± 175.7	<0.001
Whole grain (servings/day)	1.3 ± 1.0	1.1 ± 0.8	1.2 ± 0.8	1.2 ± 0.8	1.2 ± 0.8	<0.001
Dairy (cups/day)	1.2 ± 1.2	1.3 ± 1.1	1.4 ± 1.1	1.5 ± 1.1	1.6 ± 1.2	<0.001
Coffee (g/day)	762.0 ± 792.5	843.7 ± 769.7	869.5 ± 795.7	854.4 ± 804.1	846.1 ± 829.8	<0.001
Tea (g/day)	285.7 ± 525.1	259.2 ± 458.5	253.8 ± 446.9	262.9 ± 457.9	270.6 ± 486.8	<0.001
Nutrient intake						
Dietary fiber (g/day)	19.4 ± 10.5	17.1 ± 8.2	17.8 ± 8.3	18.5 ± 8.2	19.4 ± 8.4	<0.001
Saturated fatty acids (g/day)	16.6 ± 10.8	16.8 ± 9.8	20.2 ± 11.1	21.9 ± 11.7	26.2 ± 13.2	<0.001
Added sugar (tsp/day)	9.4 ± 8.8	10.0 ± 7.5	12.2 ± 8.4	13.6 ± 8.6	17.8 ± 10.5	<0.001
Sodium (mg/day)	2677.4 ± 1294.6	2496.7 ± 1103.2	2767.4 ± 1193.2	2894.8 ± 1232.1	3064.3 ± 1292.8	<0.001
Magnesium (mg/day)	328.7 ± 146.3	304.6 ± 122.1	321.2 ± 125.6	332.0 ± 125.9	351.6 ± 131.7	<0.001
Potassium (mg/day)	3287.9 ± 1418.2	3095.1 ± 1210.1	3252.6 ± 1233.5	3348.8 ± 1243.7	3476.2 ± 1286.0	<0.001
Calcium (mg/day)	722.2 ± 419.2	695.5 ± 386.7	753.7 ± 400.0	786.8 ± 404.8	843.1 ± 425.6	<0.001

^a^Values are mean ± standard deviation or counts (percentage) as indicated.

^b^“Others” refers to Asian, Pacific Islander, or American Indian.

^c^Total time of moderate to vigorous physical activities per week.

### Chocolate consumption and all-cause and cause-specific mortality

During a mean (standard deviation) follow-up length of 13.5 (3.3) years (1238513.2 person-years), a total of 19586 all-cause deaths were observed, of which 5490 (28.0%) and 6175 (31.5%) were classified as deaths from CVD and cancer, respectively. Crude mortality rates of all causes, CVD, and cancer were 158.14, 44.33, and 49.86 per 10000 person-years, respectively. Compared with no regular chocolate consumption, the maximally adjusted HRs of all-cause mortality were 0.89 (95% CI 0.84–0.94), 0.84 (95% CI 0.79–0.90), 0.86 (95% CI 0.81–0.93), and 0.87 (95% CI 0.82–0.93) for >0–0.5 servings/week, >0.5–1 serving/week, >1–2 servings/week, and >2 servings/week, respectively (*P*_trend_ = 0.009) (Table 2). Interestingly, chocolate consumption was also found to be inversely associated with cardiovascular mortality, with somewhat stronger magnitude of the association observed, while no significant association was observed for cancer mortality. In addition, we explored the association of chocolate consumption with the risk of mortality from cerebrovascular, respiratory, and Alzheimer’s diseases. In the fully adjusted model, chocolate consumption was found to be inversely associated with the risk of mortality from Alzheimer’s disease (HR for >2 vs. 0 servings/week 0.69; 95% CI 0.49–0.99) but not cerebrovascular (HR for >2 vs. 0 servings/week 0.97; 95% CI 0.73–1.28) and respiratory (HR for >2 vs. 0 servings/week 0.90; 95% CI 0.74–1.10) diseases (Table 2).

**Table 2:** Association of chocolate consumption with all-cause and cause-specific mortality^a^.

**Causes of mortality**	**Energy-adjusted chocolate consumption (servings/week)**					***P*_trend_**
	**0**	**>0–0.5**	**>0.5–1**	**>1–2**	**>2**	
**All causes**						
No. of deaths	1455	8617	3049	2187	4278	
Death rate^b^	207.00	156.44	152.07	147.94	159.00	
Model 1^c^	1.00 (reference)	0.81 (0.77–0.86)	0.77 (0.73–0.83)	0.78 (0.73–0.83)	0.82 (0.77–0.87)	**0.002**
Model 2^d^	1.00 (reference)	0.89 (0.84–0.94)	0.84 (0.79–0.90)	0.87 (0.81–0.93)	0.88 (0.83–0.94)	**0.026**
Model 3^e^	1.00 (reference)	0.89 (0.84–0.94)	0.84 (0.79–0.90)	0.86 (0.81–0.93)	0.87 (0.82–0.93)	**0.009**
**Cardiovascular disease**						
No. of deaths	483	2408	856	584	1159	
Death rate^b^	68.72	43.72	42.69	39.50	43.08	
Model 1^c^	1.00 (reference)	0.70 (0.63–0.77)	0.67 (0.60–0.75)	0.64 (0.57–0.73)	0.69 (0.62–0.76)	**0.001**
Model 2^d^	1.00 (reference)	0.79 (0.72–0.88)	0.76 (0.68–0.85)	0.77 (0.68–0.87)	0.79 (0.70–0.88)	**0.027**
Model 3^e^	1.00 (reference)	0.79 (0.72–0.88)	0.76 (0.68–0.86)	0.77 (0.68–0.87)	0.78 (0.70–0.88)	**0.020**
**Cancer**						
No. of deaths	388	2659	993	738	1397	
Death rate^b^	55.20	48.27	49.52	49.92	51.92	
Model 1^c^	1.00 (reference)	0.93 (0.84–1.04)	0.94 (0.84–1.06)	0.97 (0.86–1.10)	1.00 (0.89–1.12)	0.105
Model 2^d^	1.00 (reference)	0.95 (0.86–1.06)	0.95 (0.84–1.07)	0.99 (0.87–1.12)	0.98 (0.87–1.10)	0.595
Model 3^e^	1.00 (reference)	0.95 (0.86–1.06)	0.95 (0.84–1.07)	0.99 (0.87–1.13)	0.99 (0.88–1.11)	0.431
**Cerebrovascular disease**						
No. of deaths	74	518	177	133	224	
Death rate^b^	10.53	9.40	8.83	9.00	8.33	
Model 1^c^	1.00 (reference)	0.94 (0.74–1.20)	0.88 (0.67–1.15)	0.93 (0.70–1.23)	0.84 (0.64–1.09)	0.145
Model 2^d^	1.00 (reference)	1.04 (0.81–1.33)	0.98 (0.74–1.30)	1.10 (0.82–1.47)	0.98 (0.74–1.29)	0.503
Model 3^e^	1.00 (reference)	1.05 (0.82–1.35)	0.99 (0.75–1.31)	1.10 (0.82–1.48)	0.97 (0.73–1.28)	0.359
**Respiratory disease**						
No. of deaths	138	899	309	224	468	
Death rate^b^	19.63	16.32	15.41	15.15	17.39	
Model 1^c^	1.00 (reference)	0.89 (0.74–1.06)	0.82 (0.67–1.00)	0.83 (0.67–1.03)	0.93 (0.77–1.12)	0.284
Model 2^d^	1.00 (reference)	0.97 (0.80–1.16)	0.87 (0.71–1.06)	0.91 (0.73–1.13)	0.93 (0.77–1.14)	0.904
Model 3^e^	1.00 (reference)	0.95 (0.79–1.15)	0.85 (0.69–1.04)	0.89 (0.72–1.11)	0.90 (0.74–1.10)	0.658
**Alzheimer’s disease**						
No. of deaths	45	267	82	56	134	
Death rate^b^	6.40	4.85	4.09	3.79	4.98	
Model 1^c^	1.00 (reference)	0.74 (0.54–1.02)	0.61 (0.43–0.88)	0.59 (0.40–0.87)	0.75 (0.54–1.06)	0.088
Model 2^d^	1.00 (reference)	0.76 (0.55–1.05)	0.62 (0.42–0.89)	0.56 (0.38–0.84)	0.71 (0.50–1.01)	**0.031**
Model 3^e^	1.00 (reference)	0.78 (0.56–1.08)	0.63 (0.43–0.91)	0.57 (0.38–0.86)	0.69 (0.49–0.99)	**0.041**

^a^Values are hazard ratios (95% confidence intervals).

^b^Crude death rate per 10000 person-years.

^c^Adjusted for age (years), sex (male, female), and ethnicity (non-Hispanic white, non-Hispanic black, Hispanic, others).

^d^Adjusted for model 1 plus educational level (college below, college graduate, postgraduate), marital status (married or living as married, widowed, divorced, separated, never married), study center (10 categories), history of hypertension (yes, no), history of diabetes (yes, no), aspirin use (yes, no), hormone use status (current, former, never) for women, smoking status (current, former, never), alcohol consumption (g/day), body mass index (kg/m^2^), physical activity (min/week), and energy intake from diet (kcal/day). For all-cause and cancer mortality, the model 2 was further adjusted for family history of cancer (yes, no).

^e^Adjusted for model 2 plus consumption of red meat (g/day), processed meat (g/day), fruit (g/day), vegetable (g/day), whole grain (servings/day), dairy (cups/day), coffee (g/day), and tea (g/day).

### Subgroup analyses

A significant interaction between chocolate consumption and smoking status was observed for mortality from all causes (*P*_interaction_ = 0.006) and CVD (*P*_interaction_ = 0.045) but not cancer (*P*_interaction_ = 0.504) (Table 3). Specifically, the favorable association between chocolate consumption and mortality from all causes and CVD was found to be more pronounced in never smokers than in current or former smokers. No significant interaction was observed for remaining stratification factors, namely age, sex, trial group, BMI, alcohol consumption, milk consumption, and history of hypertension. In addition, we performed a subgroup analysis by smoking status for the association of chocolate consumption with the risk of mortality from Alzheimer’s disease; the results showed that there was no significant difference in the risk of mortality from Alzheimer’s disease between current or past and never smokers (*P*_interaction_ = 0.812).

**Table 3:** Subgroup analyses on the association of chocolate consumption with mortality from all causes, cardiovascular disease, and cancer^a^.

**Subgroup variable**	**Energy-adjusted chocolate consumption (servings/week)**					***P*_interaction_**
	**0**	**>0–0.5**	**>0.5–1**	**>1–2**	**>2**	
**All-cause mortality**						
Age (years)						
≥60	1.00 (reference)	0.88 (0.83–0.93)	0.83 (0.77–0.88)	0.85 (0.80–0.92)	0.87 (0.81–0.93)	0.369
<60	1.00 (reference)	1.03 (0.83–1.28)	1.03 (0.81–1.31)	1.01 (0.79–1.30)	0.96 (0.75–1.21)	
Sex						
Male	1.00 (reference)	0.90 (0.83–0.98)	0.86 (0.79–0.95)	0.87 (0.79–0.96)	0.90 (0.82–0.98)	0.879
Female	1.00 (reference)	0.87 (0.80–0.94)	0.82 (0.75–0.89)	0.85 (0.77–0.94)	0.84 (0.77–0.92)	
Trial group						
Screening	1.00 (reference)	1.00 (0.94–1.07)	0.89 (0.83–0.95)	0.95 (0.90–1.02)	0.93 (0.87–1.00)	0.683
Control	1.00 (reference)	0.97 (0.91–1.04)	0.88 (0.82–0.94)	0.92 (0.87–0.98)	0.94 (0.88–1.00)	
Body mass index (kg/m^2^)						
≥25	1.00 (reference)	0.88 (0.82–0.95)	0.86 (0.79–0.93)	0.86 (0.79–0.94)	0.88 (0.81–0.95)	0.755
<25	1.00 (reference)	0.90 (0.82–0.99)	0.82 (0.74–0.92)	0.89 (0.79–1.00)	0.88 (0.79–0.98)	
Alcohol consumption (g/day)^b^						
No, light or moderate	1.00 (reference)	0.91 (0.85–0.96)	0.85 (0.79–0.91)	0.87 (0.81–0.94)	0.88 (0.82–0.94)	0.306
Heavy	1.00 (reference)	0.79 (0.68–0.93)	0.80 (0.67–0.95)	0.86 (0.71–1.03)	0.83 (0.69–0.98)	
Smoking status						
Current or former	1.00 (reference)	0.91 (0.85–0.98)	0.92 (0.84–0.99)	0.93 (0.85–1.01)	0.94 (0.87–1.02)	**0.006**
Never	1.00 (reference)	0.87 (0.79–0.95)	0.76 (0.69–0.85)	0.80 (0.72–0.89)	0.81 (0.74–0.90)	
Milk consumption (servings/day)						
≥ median	1.00 (reference)	0.91 (0.83–0.99)	0.84 (0.77–0.93)	0.88 (0.79–0.97)	0.85 (0.78–0.93)	0.118
< median	1.00 (reference)	0.87 (0.80–0.94)	0.84 (0.77–0.92)	0.86 (0.78–0.94)	0.91 (0.83–1.00)	
History of hypertension						
Yes	1.00 (reference)	0.92 (0.85–1.01)	0.86 (0.77–0.95)	0.94 (0.84–1.05)	0.93 (0.84–1.03)	0.207
No	1.00 (reference)	0.86 (0.79–0.92)	0.83 (0.76–0.90)	0.82 (0.75–0.89)	0.83 (0.77–0.90)	
**Cardiovascular mortality**						
Age (years)						
≥60	1.00 (reference)	0.80 (0.72–0.89)	0.78 (0.69–0.88)	0.77 (0.68–0.88)	0.79 (0.70–0.89)	0.639
<60	1.00 (reference)	0.73 (0.50–1.05)	0.59 (0.38–0.92)	0.68 (0.43–1.09)	0.76 (0.50–1.15)	
Sex						
Male	1.00 (reference)	0.81 (0.70–0.94)	0.84 (0.71–0.99)	0.80 (0.67–0.96)	0.83 (0.70–0.98)	0.345
Female	1.00 (reference)	0.77 (0.67–0.89)	0.68 (0.58–0.80)	0.73 (0.61–0.87)	0.74 (0.63–0.86)	
Trial group						
Screening	1.00 (reference)	0.98 (0.87–1.11)	0.88 (0.77–1.00)	0.87 (0.77–0.98)	0.90 (0.80–1.02)	0.627
Control	1.00 (reference)	0.91 (0.81–1.02)	0.83 (0.73–0.94)	0.91 (0.80–1.02)	0.89 (0.78–1.01)	
Body mass index (kg/m^2^)						
≥25	1.00 (reference)	0.81 (0.72–0.91)	0.77 (0.67–0.88)	0.77 (0.66–0.89)	0.81 (0.70–0.93)	0.891
<25	1.00 (reference)	0.78 (0.65–0.93)	0.79 (0.64–0.97)	0.79 (0.63–0.99)	0.76 (0.62–0.93)	
Alcohol consumption (g/day)^b^						
No, light or moderate	1.00 (reference)	0.91 (0.85–0.96)	0.85 (0.79–0.91)	0.87 (0.81–0.93)	0.88 (0.82–0.94)	0.279
Heavy	1.00 (reference)	0.80 (0.68–0.93)	0.80 (0.67–0.95)	0.86 (0.71–1.04)	0.83 (0.70–0.99)	
Smoking status						
Current or former	1.00 (reference)	0.79 (0.69–0.90)	0.82 (0.70–0.95)	0.83 (0.70–0.97)	0.86 (0.74–1.00)	**0.045**
Never	1.00 (reference)	0.81 (0.70–0.95)	0.71 (0.60–0.85)	0.71 (0.58–0.86)	0.72 (0.60–0.86)	
Milk consumption (servings/day)						
≥ median	1.00 (reference)	0.88 (0.76–1.03)	0.80 (0.68–0.95)	0.80 (0.67–0.96)	0.84 (0.71–0.99)	0.227
< median	1.00 (reference)	0.72 (0.63–0.83)	0.74 (0.63–0.87)	0.75 (0.63–0.89)	0.75 (0.64–0.88)	
History of hypertension						
Yes	1.00 (reference)	0.85 (0.73–0.98)	0.81 (0.68–0.96)	0.85 (0.70–1.02)	0.84 (0.71–1.00)	0.636
No	1.00 (reference)	0.75 (0.65–0.86)	0.73 (0.62–0.85)	0.70 (0.60–0.83)	0.73 (0.63–0.85)	
**Cancer mortality**						
Age (years)						
≥60	1.00 (reference)	0.94 (0.84–1.06)	0.92 (0.81–1.04)	0.97 (0.85– 1.11)	0.98 (0.87–1.11)	0.348
<60	1.00 (reference)	1.05 (0.73–1.50)	1.24 (0.84–1.82)	1.19 (0.80– 1.77)	1.05 (0.71–1.54)	
Sex						
Male	1.00 (reference)	1.07 (0.91–1.26)	1.05 (0.88–1.25)	1.14 (0.95–1.37)	1.10 (0.92–1.31)	0.339
Female	1.00 (reference)	0.86 (0.74–1.00)	0.86 (0.73–1.01)	0.87 (0.73–1.04)	0.90 (0.76–1.05)	
Trial group						
Screening	1.00 (reference)	0.92 (0.81–1.03)	0.93 (0.83–1.06)	0.99 (0.89–1.11)	1.06 (0.94–1.20)	0.265
Control	1.00 (reference)	0.95 (0.84–1.06)	0.99 (0.88–1.11)	0.92 (0.82–1.03)	0.95 (0.84–1.08)	
Body mass index (kg/m^2^)						
≥25	1.00 (reference)	1.01 (0.88–1.16)	1.01 (0.87–1.17)	1.06 (0.91–1.24)	1.01 (0.87–1.17)	0.305
<25	1.00 (reference)	0.86 (0.72–1.02)	0.84 (0.69–1.04)	0.87 (0.70–1.08)	0.95 (0.78–1.16)	
Alcohol consumption (g/day)^a^						
No, light or moderate	1.00 (reference)	1.02 (0.90–1.15)	1.00 (0.88–1.14)	1.05 (0.92–1.21)	1.05 (0.92–1.20)	0.108
Heavy	1.00 (reference)	0.69 (0.54–0.88)	0.72 (0.54–0.96)	0.73 (0.54–1.00)	0.71 (0.53–0.95)	
Smoking status						
Current or former	1.00 (reference)	0.99 (0.87–1.13)	0.98 (0.84–1.13)	1.08 (0.92–1.26)	1.04 (0.90–1.20)	0.504
Never	1.00 (reference)	0.91 (0.76–1.09)	0.94 (0.76–1.15)	0.88 (0.71–1.10)	0.96 (0.78–1.17)	
Milk consumption (servings/day)						
≥ median	1.00 (reference)	1.02 (0.87–1.20)	1.01 (0.84–1.20)	1.06 (0.88–1.27)	0.98 (0.82–1.17)	0.117
< median	1.00 (reference)	0.90 (0.78–1.04)	0.90 (0.76–1.06)	0.94 (0.79–1.12)	1.03 (0.88–1.22)	
History of hypertension						
Yes	1.00 (reference)	1.09 (0.90–1.31)	1.02 (0.82–1.26)	1.21 (0.97–1.51)	1.13 (0.92–1.39)	0.204
No	1.00 (reference)	0.89 (0.78–1.02)	0.91 (0.78–1.05	0.90 (0.77–1.05)	0.92 (0.80–1.06)	

^a^Values are hazard ratios (95% confidence intervals). Hazard ratios were adjusted for age (years), sex (male, female), ethnicity (non-Hispanic white, non-Hispanic black, Hispanic, others), educational level (college below, college graduate, postgraduate), marital status (married or living as married, widowed, divorced, separated, never married), study center (10 categories), history of hypertension (yes, no), history of diabetes (yes, no), aspirin use (yes, no), hormone use status (current, former, never) for women, smoking status (current, former, never), alcohol consumption (g/day), body mass index (kg/m^2^), physical activity (min/week), energy intake from diet (kcal/day), and consumption of red meat (g/day), processed meat (g/day), fruit (g/day), vegetable (g/day), whole grain (servings/day), dairy (cups/day), coffee (g/day), and tea (g/day). For all-cause and cancer mortality, the model was further adjusted for family history of cancer (yes, no). In subgroup analyses stratified by sex, smoking status, and history of hypertension, hazard ratios were not adjusted for the stratification factor.

^b^For men, light, moderate, and heavy alcohol consumption referred to ≤ 6 g/day, > 6–28 g/day, and > 28 g/day, respectively; for women, light, moderate, and heavy alcohol consumption referred to ≤ 6 g/day, > 6–14 g/day, and > 14 g/day, respectively.

### Dose–response analyses

**Figure 1:**
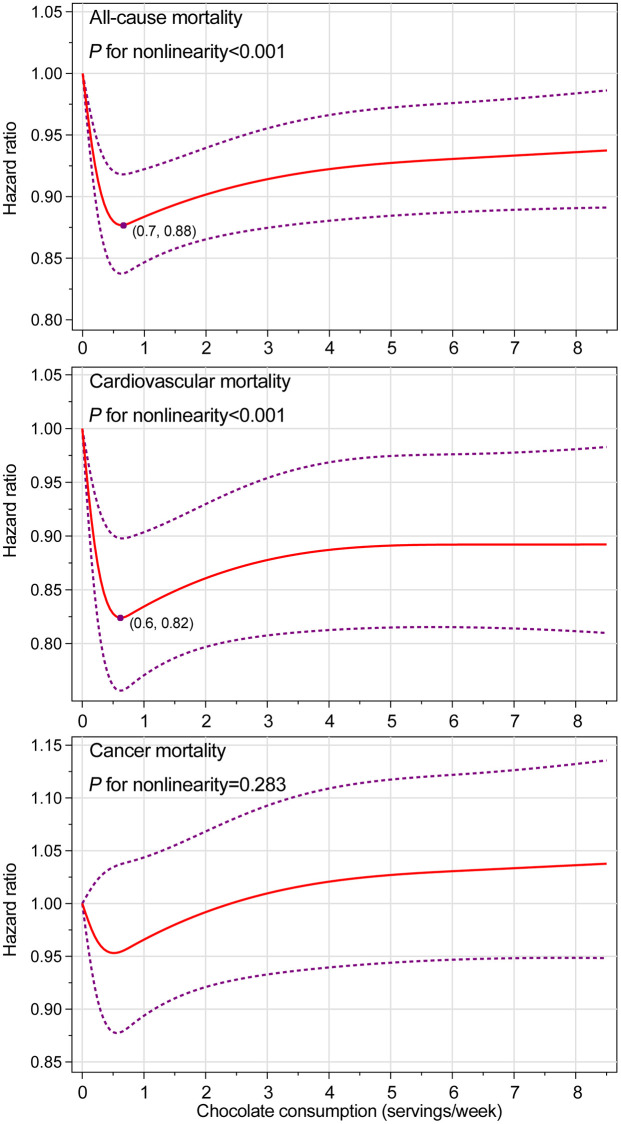
**Nonlinear dose–response analyses on energy-adjusted chocolate consumption and mortality from all causes, cardiovascular disease, and cancer in the whole study population.** The reference level was set at 0 servings/week. Hazard ratio was adjusted for age, sex, ethnicity, educational level, marital status, study center, history of hypertension, history of diabetes, aspirin use, hormone use status for women, smoking status, alcohol consumption, body mass index, physical activity, energy intake from diet, and consumption of red meat, processed meat, fruit, vegetable, whole grain, dairy, coffee, and tea. For all-cause and cancer mortality, the hazard ratio was further adjusted for family history of cancer. The red solid line represents the fitted nonlinear trend, and the purple short-dash line represents corresponding 95% confidence interval.

In the entire study population, chocolate consumption was found to be associated with lower risks of death from all causes (*P*_nonlinearity_ <0.001) and CVD (*P*_nonlinearity_ <0.001) in a nonlinear dose–response pattern, with the lowest risk observed at chocolate consumption of 0.7 servings/week and 0.6 servings/week, respectively (Figure 1). Given the aforementioned significant interaction between chocolate consumption and smoking status, we further conducted smoking status-specific dose–response analyses (Supplementary Figures 1 and 2 for never and current or former smokers, respectively). The nonlinear dose–response associations of chocolate consumption with risks of death from all causes and CVD were only found among never smoker, with the lowest risk observed at chocolate consumption of 0.6 servings/week and 0.7 servings/week, respectively. No significant dose–response association was found for cancer mortality in the entire study population as well as in never or current or former smokers.

### Sensitivity analyses

The initial associations of chocolate consumption with risks of death from all causes, CVD, and cancer did not change materially in a large range of sensitivity analyses (Supplementary Table 1).

## DISCUSSION

In this post hoc analysis, chocolate consumption was found to be inversely associated with all-cause and cardiovascular mortality in a nonlinear dose–response manner, with the lowest risk observed at chocolate consumption of 0.7 servings/week and 0.6 servings/week, respectively, whereas no significant association was found for cancer mortality. Subgroup analyses further found that the inverse association of chocolate consumption with all-cause and cardiovascular mortality was more pronounced in never smokers. In addition, chocolate consumption was found to be inversely associated with the risk of death from Alzheimer’s disease but not cerebrovascular and respiratory diseases.

To date, only a small number of studies have determined the potential association between chocolate consumption and mortality [9, 16]. A cohort study of 1169 acute myocardial infarction survivors [16] observed that higher chocolate consumption conferred lower mortality from CVD but not all causes. Subsequently, a prospective study of 84709 postmenopausal women revealed an inverse association between chocolate consumption and mortality from all causes, CVD, and Alzheimer’s disease [9]. However, whether these observations can be extended to the general population is unknown. Based on prospective data from the PLCO Cancer Screening Trial, we found that chocolate consumption was inversely associated with risks of death from all causes, cardiovascular disease, and Alzheimer’s disease, which is consistent with the results from the study by Sun et al. [9]. Our study presents the following important information. First, our study provided data on the association of chocolate consumption with cancer mortality. Second, our study identified the shape and the nadir of dose–response curve for the chocolate-mortality association, which are essential for recommending optimal chocolate consumption to the public. Third, our study found that smoking behavior could significantly modify the observed inverse association of chocolate consumption with all-cause and cardiovascular mortality.

Previously, a prospective study of healthy men and women found that increasing chocolate consumption was monotonically associated with decreased cardiovascular mortality (*P*_linearity_ = 0.011) [10]. In contrast, our dose–response analysis found that the association of chocolate consumption with cardiovascular mortality was nonlinear, with the lowest risk observed at 0.6 servings/week, indicating that individuals consuming low amounts of chocolate could benefit most. This finding can be explained by the fact that chocolate is rich in sugar, carbohydrate, and fat [17]. High intakes of these macronutrients are associated with increased mortality [18–20], which possibly weakens or even negates the favorable association between chocolate and mortality. In fact, several prospective studies also found that low to moderate but not high chocolate consumption was favorably associated with risks of atrial fibrillation [21] and heart failure [22, 23].

In our study, the information on chocolate types, such as milk chocolate and dark chocolate, was not collected by the DHQ. However, it should be noted that different types of chocolate have different nutritional ingredients [17]. For example, dark chocolate has a significantly higher proportion of cocoa solids than milk chocolate [24], resulting in that dark chocolate contains more polyphenols, a class of bioactive substances mainly found in vegetables and fruits [17]. In addition, milk is found to be capable of reducing the absorption of antioxidants from chocolate [25]. Thus, it is biologically possible that dark chocolate has stronger physiological effects in human body than milk chocolate. Indeed, a crossover study of 16 young men found that compared with participants consuming milk chocolate, those consuming dark chocolate felt more satiated and had lower desire to eat something sweet and lower energy intake [26]. Dark chocolate was also found to have a higher ability to increase total antioxidant capacity of human plasma than milk chocolate [25]. These facts raise a possibility that dark chocolate has more benefits in improving health outcomes than milk chocolate. Unfortunately, the unavailability of chocolate type data in our study hindered us to determine whether there is a difference in reducing mortality between dark chocolate and milk chocolate. Nonetheless, in the US, milk chocolate is more popular than dark chocolate at the time of the study [27], indicating that the observed associations with all-cause and cardiovascular mortality could be largely driven by milk chocolate. In addition, after a thorough literature review, we failed to find an observational study that has examined the potential impacts of chocolate types on health outcomes [4, 6, 15], reminding us that this seems to be a common limitation of studies in this filed. Hence, future studies should pay much attention to the assessment of chocolate types, and investigate the associations with health outcomes by specific chocolate types.

Interestingly, we observed that the inverse association of chocolate consumption with all-cause and cardiovascular mortality was more pronounced in never smokers than in current or former smokers. Similarly, Mink and colleagues also observed that the favorable association between dietary intake of flavonol, a bioactive ingredient in chocolate, and all-cause mortality was more pronounced in never smokers than in ever smokers [11]. These observations suggest that smoking behavior possibly has interactions with chocolate consumption in biological pathways. It is well established that smoking leads to increased levels of systemic inflammation and oxidative stress in human body [28, 29], resulting in that smokers have a higher risk of death than never smokers [30]. Thus, it is highly possible that the favorable association of chocolate consumption with all-cause and cardiovascular mortality has been weakened or even overcome by the above-mentioned harmful effects of smoking behavior. In addition, as mentioned above, milk has been reported to be able to interfere with the absorption of antioxidants from chocolate [25]; hence, we performed a subgroup analysis to determine whether the association of chocolate consumption with mortality could be modified by this factor. However, we did not observe an expected interaction after stratifying for milk consumption. In fact, Mostofsky and colleagues also found that the association between chocolate consumption and the risk of heart failure could not be modified by milk consumption [23]. The exact reason behind this phenomenon is unclear, but may be due to the fact that chocolate and milk are not consumed simultaneously in the daily life.

The inverse association of chocolate consumption with all-cause and cardiovascular mortality is biologically plausible. Several randomized controlled trials have found that dark chocolate consumption efficiently decreases blood pressure [31–34], increases insulin sensitivity [32, 34], improves cholesterol profile [33, 35, 36] and endothelial function [37], and inhibits platelet aggregation [38]; these cardiometabolic effects of chocolate have been suggested to mediate its favorable association with CVD risk [39, 40]. Chocolate is a significant source of flavonoids and is especially rich in proanthocyanidins, catechins, and epicatachins [41]. *In vitro* and animal studies have indicated that flavonoids have antioxidant and anti-inflammatory effects and favorable impacts on glucose-insulin homeostasis [41]. Also, dietary flavonoid intake has been identified to be associated with lower all-cause and cardiovascular mortality [42]. Therefore, the above-mentioned cardiometabolic effects of chocolate may be explained by the biological effects of flavonoids in nature, at least partly. In addition, we observed an inverse association between chocolate consumption and the risk of death from Alzheimer’s disease. In fact, previous observational studies had found that chocolate consumption was associated with a decreased risk of cognitive decline [43, 44]. The observed association of chocolate consumption with the risk of death from Alzheimer’s disease could be attributable to the neuroprotective effects of cocoa [45]. An *in vitro* Alzheimer’s disease model study suggested that cocoa exerted its neuroprotective effects through activating BDNF signaling pathway [46]. In addition, chocolate contains a certain amount of caffeine, which was found to have protective effects on mortality from all causes and CVD [47] and have some beneficial effects on the development of Alzheimer’s disease [48]; thus, the roles of caffeine in the observed inverse association should not be ignored. More studies are needed to determine which ingredients in chocolate are actually responsible for the inverse association we observed.

Our study has several limitations that should be acknowledged and discussed. First, chocolate consumption was only assessed at baseline in the PLCO Cancer Screening Trial by the DHQ. The assessment of dietary exposure at one time point possibly results in non-differential bias, considering that dietary habits could change over time. Nonetheless, a classic assumption in nutritional epidemiology is that an adult’s dietary habits would not change dramatically during several years. Moreover, it has been suggested that the method only using baseline diet generally yields a weaker association than that using the cumulative averages [49]. Second, in the present study, we used death certificates to obtain the underlying causes of death. However, it should be highlighted that the cause of death from death certificates is misclassified in some conditions, especially deaths due to respiratory or digestive diseases [50]. Hence, our results on chocolate consumption and cause-specific mortality might be subject to misclassification bias. Third, although we had controlled for a wide range of possible confounders, our results could be susceptible to residual confounding because of the observational design of this study. Fourth, given that CVD mortality was a primary outcome in our study, we excluded subjects with a history of acute myocardial infarction or stroke at baseline; however, as the PLCO Cancer Screening Trial did not collect data on coronary artery disease, thus we could not exclude subjects with this disease. Hence, our results might be subject to reverse causation and confounded by other CVDs (e.g., coronary artery disease). Fifth, chocolate consumption was assessed by a self-administrated food frequency questionnaire in our study. Thus, the assessment of chocolate consumption might be subject to measurement errors. Nonetheless, this bias is nondifferential, given that it is not expected to be associated with the future risk of death, and thus tends to attenuate the association of our interest. Moreover, chocolate consumption might have been underestimated in our study as it might be added to other foods. Finally, our findings derived from a US population aged 55 to 74 years with a relatively high chocolate consumption, and thus might not be extended to other age groups or other populations with lower chocolate consumption.

In conclusion, in this US population aged 55 to 74 years, chocolate consumption is associated with lower risks of death from all causes, cardiovascular disease, and Alzheimer’s disease. Due to the observational design of our study, these findings do not imply causation and needed to be further confirmed in other populations and settings. If confirmed, eating chocolate may be a good choice for improving longevity. Future studies are warranted to clarify the potential influence of chocolate types on the observed association.

## MATERIALS AND METHODS

This study was reported following the Strengthening the Reporting of Observational Studies in Epidemiology statement [51].

### Study population

A total of 76682 men and 78215 women aged 55 to 74 years were enrolled to the PLCO Cancer Screening Trial between November 1993 and September 2001 in ten screening centers across the US (Washington, Pittsburgh, Honolulu, Denver, Marshfield, Minneapolis, Birmingham, Salt Lake City, Detroit, and St Louis). The PLCO Cancer Screening Trial was a multicenter randomized controlled trial designed to determine whether screening for prostate, lung, colorectal, and ovarian cancers could reduce mortality from these cancers, and its study design and implementation were described elsewhere [52]. The PLCO Cancer Screening Trial was approved by the Institutional Review Boards of the US National Cancer Institute and each study center, and written informed consent was obtained from all individuals. The study was conducted in accordance with the Declaration of Helsinki.

**Figure 2:**
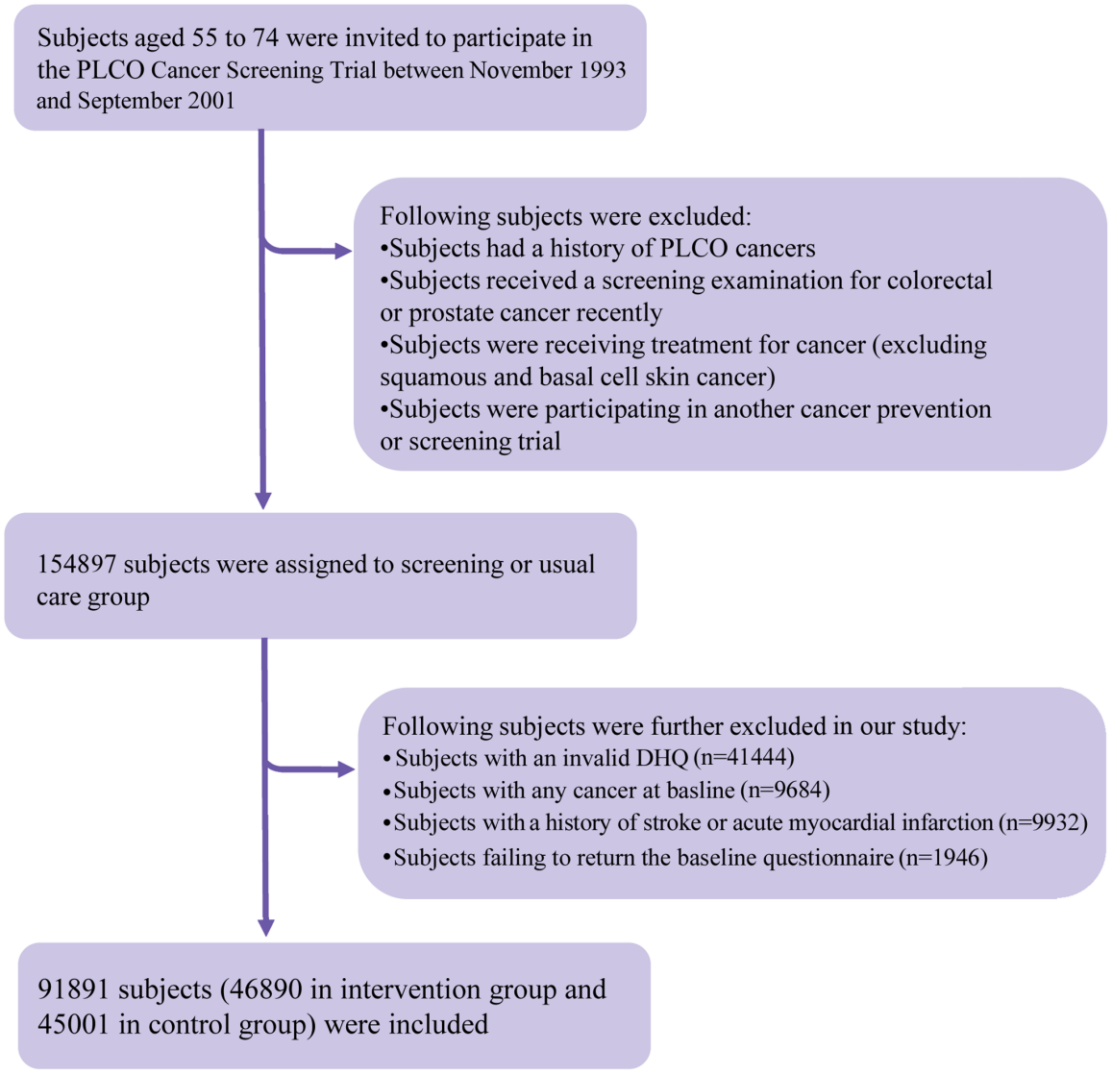
**The study flow chart of identifying eligible participants.** The percentage in the figure was calculated as the number of participants excluded by each exclusion criterion divided by the total number of participants in the PLCO Cancer Screening Trial (i.e., 154897). PLCO, Prostate, Lung, Colorectal, and Ovarian; DHQ, diet history questionnaire.

**Figure 3:**
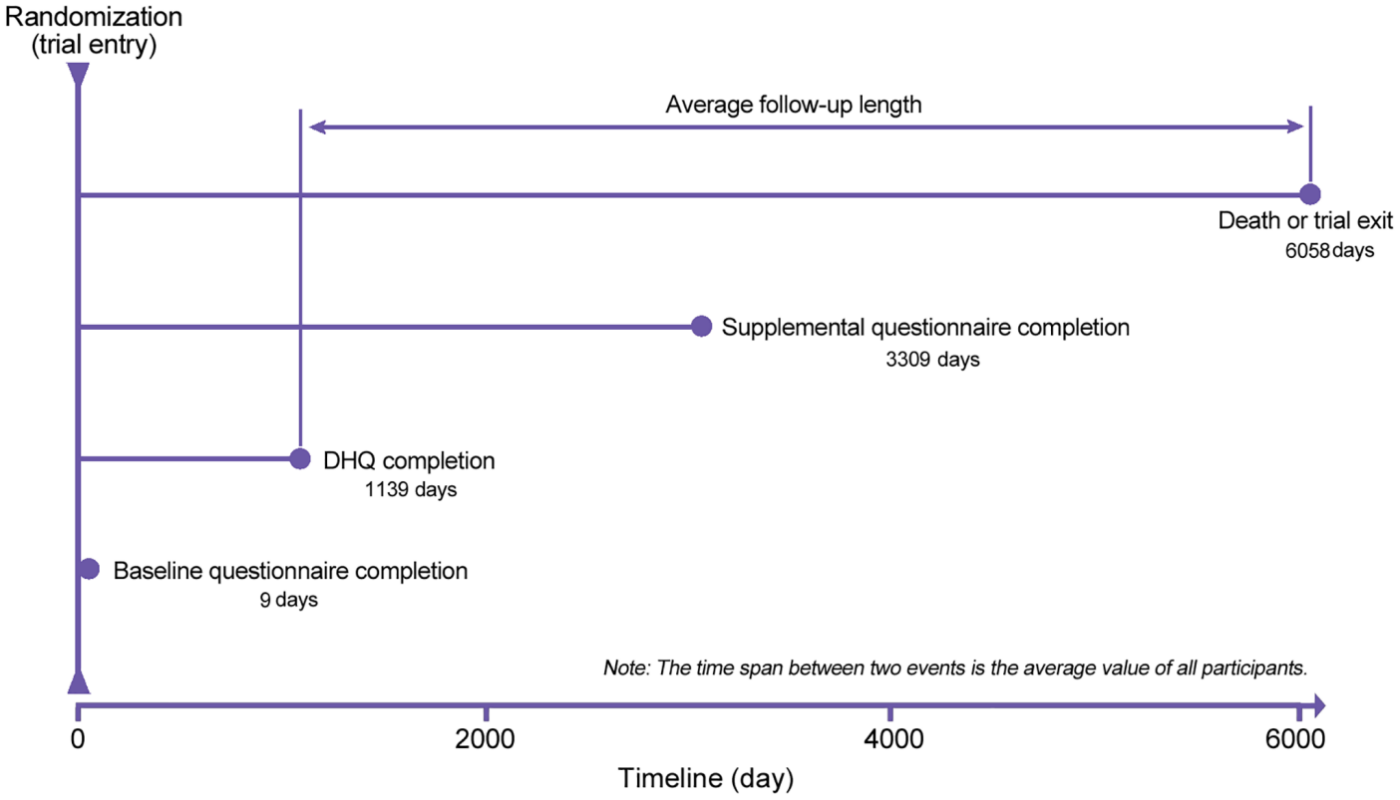
The timeline and follow-up scheme of the present study. DHQ, diet history questionnaire.

The following individuals were further excluded from our study: (1) individuals with an invalid diet history questionnaire (DHQ), which refers to missing the date of DHQ completion, missing ≥8 DHQ items, death date prior to DHQ completion date, or the presence of extreme values of energy intake (i.e., top 1% or bottom 1%) (*n* = 41444); (2) individuals diagnosed with any cancer before baseline questionnaire or DHQ completion (*n* = 9684); (3) individuals having a history of acute myocardial infarction or stroke at baseline (*n* = 9932); and (4) individuals failing to return the baseline questionnaire (*n* = 1946). After exclusions, a total of 91891 individuals were included (Figure 2). Importantly, no marked differences were observed in sociodemographic characteristics and medical histories between included and excluded populations (all standardized differences <0.1; Supplementary Table 2), suggesting a small possibility of selection bias due to the exclusion of a large number of individuals in our study. For all eligible individuals, follow-up length was computed from the date of DHQ completion to the date of death, loss to follow-up, the date of mortality, or the end of follow-up (i.e., December 31, 2015), whichever occurred first (Figure 3).

### Assessment of chocolate consumption

Chocolate consumption was evaluated using the above-mentioned DHQ in the present study. Participants were asked to answer “How often did you eat chocolate during the past year?”. There were 11 predefined answers: never, 1–6 times/year, 7–11 times/year, 1 time/month, 2–3 times/month, 1 time/week, 2 times/week, 3–4 times/week, 5–6 times/week, 1 time/day, and ≥2 times/day. Chocolate consumers were further asked to answer “Each time you ate chocolate, how much did you usually eat?”. The predefined answers were “Less than 1 average bar or less than 28.35 g”, “1 average bar or 28.35 to 56.70 g”, and “More than 1 average bar or more than 56.70 g”. Chocolate consumption (servings/week, 1 serving size of chocolate was defined as 28.35g chocolate [53]) was estimated by multiplying the frequency of chocolate consumption by portion size. Notably, chocolate consumption involved in all analyses was adjusted for energy intake with the residual method for removing extraneous variation of chocolate consumption resulting from energy intake [54].

### Outcome assessment

In the PLCO Cancer Screening Trial, a mailed annual study update form was used to confirm participants’ vital status. For participants failing to return this form, they were repeatedly contacted through telephone or e-mail. Additionally, the information on death was adjudicated by periodic linkage to the National Death Index for increasing its completeness. The International Classification of Diseases, ninth Revision (ICD-9), which was frequently used when the trial began, was used to define the underlying causes of mortality obtained from death certificates: CVD (ICD codes 390–459) and cancer (ICD codes 140–209).

### Assessment of covariates

A self-administrated baseline questionnaire was used to collect the following data: sex, marital status, ethnicity, height, body weight, educational degree, family history of cancer, history of diabetes, history of hypertension, aspirin use, and smoking status. Body mass index (BMI) was computed as body weight (kg) divided by height squared (m^2^). A DHQ (version 1.0, National Cancer Institute, 2007) was used to assess the remaining covariates: age at DHQ completion, alcohol consumption, energy intake from diet, food consumption, and nutrient intake. The DHQ was a self-administered 137-item food frequency questionnaire, which was developed to evaluate the frequency and portion size of food consumption and nutrient intake over the past 12 months. Notably, the validity of the DHQ had been confirmed against four 24-h dietary recalls in The Eating at America's Table Study [55]. Daily food consumption was estimated by multiplying the food frequency by portion size; daily nutrient intake was estimated based on the Nutrition Data Systems for Research [56] and the USDA's 1994-96 Continuing Survey of Food Intakes by Individuals [57]. Healthy Eating Index-2015, an indicator of diet quality, with higher scores indicating higher diet quality, was computed as previously described [58]. Physical activity level was approximated based on the frequency and duration of moderate and strenuous activities, which were collected with a self-administrated supplemental questionnaire. The supplemental questionnaire was introduced in 2006 in the PLCO Cancer Screening Trial; in our study, the mean (standard deviation) time from trial entry to the completion of this questionnaire was 3309 (680) days (Figure 3).

### Statistical analysis

As there were several variables with missing data (see Supplementary Table 3), for reducing selection bias and increasing statistical power, we assumed that these data were missing at random, and then used multiple imputation with chained equations to impute missing data (the number of imputations = 25) [59]. All variables involved in statistical analyses were used to produce the imputed data sets.

Cox proportional hazards regression was performed to estimate hazard ratios (HRs) and 95% confidence intervals (CIs) for the association of chocolate consumption with all-cause and cause-specific mortality, with person-year as time variable. No evidence for the violation of proportional hazards assumption was detected using Schoenfeld residuals. Chocolate consumption was classified into five categories based on its distribution in our study population and published articles on chocolate consumption and health outcomes [6, 23, 60] (0, >0–0.5, >0.5–1, >1–2, >2 servings/week), with 0 servings/week as the reference group. A *P* for quadratic trend was obtained by assigning the median value of each category to each individual in the category, and then tested the statistical significance of its squared value in regression analyses. Covariate included in multivariable analyses was selected based on the change-in-estimate approach [61] and the existing literature. Specifically, model 1 was adjusted for age, sex, and ethnicity; model 2 was further adjusted for educational degree, marital status, study center, history of hypertension, history of diabetes, aspirin use, hormone use status for women, smoking status, alcohol consumption, BMI, physical activity, and energy intake from diet; and model 3 was further adjusted for consumption of red meat, processed meat, fruit, vegetable, whole grain, dairy, coffee, and tea. Of note, for all-cause and cancer mortality, model 2 was additionally adjusted for family history of cancer.

Prespecified subgroup analyses were performed after stratifying for age (≥ 65 vs. < 65 years), sex (male vs. female), trial group (screening group vs. control group), BMI (≥ 25 vs. < 25 kg/m^2^) [62, 63], alcohol consumption (no, light, or moderate vs. heavy), smoking status (current or former vs. never), milk consumption (≥ median vs. < median), and history of hypertension (yes vs. no). In our study, for men, light, moderate, and heavy alcohol consumption referred to ≤ 6 g/day, > 6–28 g/day, and > 28 g/day, respectively; for women, light, moderate, and heavy alcohol consumption referred to ≤ 6 g/day, > 6–14 g/day, and > 14 g/day, respectively [64]. A *P*_interaction_ was obtained via a likelihood ratio test, in which models with and without interaction terms were compared, before conducting the above-mentioned subgroup analyses to avoid spurious subgroup differences.

Restricted cubic spline functions [65] with four knots located at the 5th, 35th, 65th, and 95th percentiles were used to describe the shape and the nadir of the dose–response curves for the association of chocolate consumption with all-cause and cause-specific mortality, with the reference level set at 0 servings/week. Of note, the choice of the number and location of knots was based on the recommendations by Harrell [66] and Akaike's information criterion [67]. Importantly, for minimizing the potential influence of extreme values, individuals with the top 2.5% of chocolate consumption (i.e., ≥ 9.1 servings/week) were excluded from the dose–response analysis. A *P*_nonlinearity_ was obtained by testing the null hypothesis that regression coefficients of the second and third splines were equal to 0 [65].

A series of sensitivity analyses were conducted to assess the stability of our results: (1) excluded individuals with extreme values of calorie intake, which were defined as <800 or >4000 kcal/day for men and <500 or >3500 kcal/day for women [68]; (2) excluded deaths observed within the first five years of follow-up to evaluate the potential influence of reverse causation; (3) adjusted for propensity score on crude model (all covariates in model 3 were used to compute propensity score for each participant with a logistic regression); (4) additionally adjusted for Healthy Eating Index-2015 to evaluate the potential influence of diet quality; (5) additionally adjusted for intakes of sodium, added sugars, and saturated fatty acids to indirectly evaluate the potential influence of adherence to a healthy eating pattern, as the Dietary Guidelines for Americans recommended that a healthy eating pattern should limit the intakes of these nutrients [69].

Continuous variables were shown as mean (standard deviation), and categorical variables were shown as counts (percentage). The ANOVA test and the χ^2^ test were employed to compare the differences of continuous and categorical variables, respectively, across the categories of chocolate consumption. Statistical analyses were performed using Stata version 12.0 (StataCorp, College Station, TX), and the corresponding results were considered statistically significant when a two-tailed *P* value was less than 0.05.

## Supplementary Materials

Supplementary Figures

Supplementary Tables
