# Supplementary material for: Chocolate consumption and all-cause and cause-specific mortality in a US population: a post hoc analysis of the PLCO cancer screening trial
Source: Aging (Albany NY). 2021 Jul 29;13(14):18564–85. doi: 10.18632/aging.203302 (PMC8351724; doi:10.18632/aging.203302)
Supplement: Supplementary Figures [file aging-13-203302-s001.pdf]

SUPPLEMENTARY FIGURES

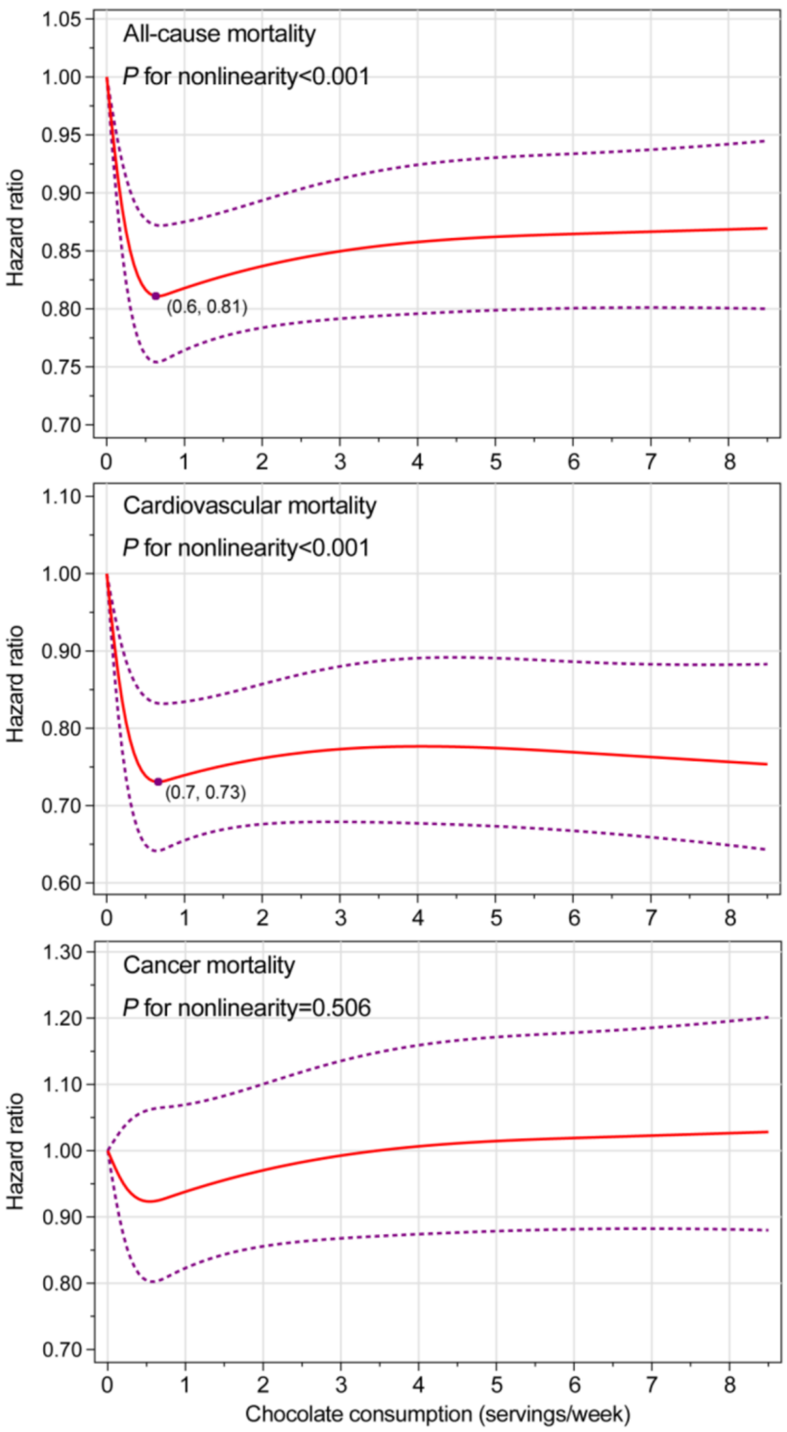

Supplementary Figure 1. Nonlinear dose–response analyses on energy-adjusted chocolate consumption and mortality from all causes, cardiovascular disease, and cancer in never smokers. The reference level was set at 0 servings/week. Hazard ratio was adjusted for age, sex, ethnicity, educational level, marital status, study center, history of hypertension, history of diabetes, aspirin use, hormone use status for women, alcohol consumption, body mass index, physical activity, energy intake from diet, and consumption of red meat, processed meat, fruit, vegetable, whole grain, dairy, coffee, and tea. For all-cause and cancer mortality, the hazard ratio was further adjusted for family history of cancer. The red solid line represents the fitted nonlinear trend, and the purple short-dash line represents corresponding 95% confidence interval.

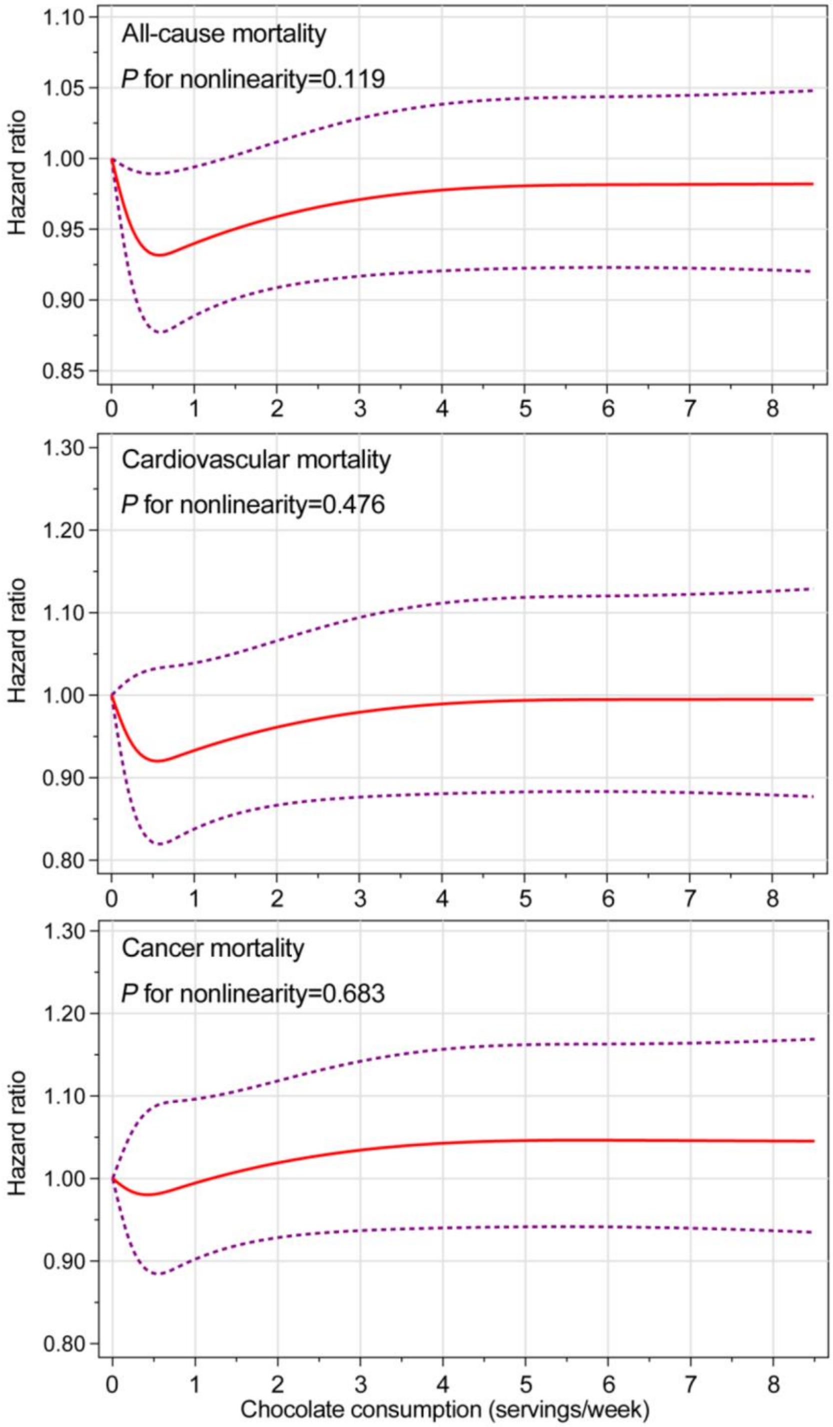

Supplementary Figure 2. Nonlinear dose–response analyses on energy-adjusted chocolate consumption and mortality from all causes, cardiovascular disease, and cancer in current or former smokers. The reference level was set at 0 servings/week. Hazard ratio was adjusted for age, sex, ethnicity, educational level, marital status, study center, history of hypertension, history of diabetes, aspirin use, hormone use status for women, alcohol consumption, body mass index, physical activity, energy intake from diet, and consumption of red meat, processed meat, fruit, vegetable, whole grain, dairy, coffee, and tea. For all-cause and cancer mortality, the hazard ratio was further adjusted for family history of cancer. The red solid line represents the fitted nonlinear trend, and the purple short-dash line represents corresponding 95% confidence interval.
