# Supplementary material for: Chocolate consumption and all-cause and cause-specific mortality in a US population: a post hoc analysis of the PLCO cancer screening trial
Source: Aging (Albany NY). 2021 Jul 29;13(14):18564–85. doi: 10.18632/aging.203302 (PMC8351724; doi:10.18632/aging.203302)
Supplement: Supplementary Tables [file aging-13-203302-s002.pdf]

SUPPLEMENTARY TABLES

Supplementary Table 1. Sensitivity analyses on the association of chocolate consumption with mortality from all causes, cardiovascular disease, and cancer[a].

Categories	Chocolate consumption (servings/week)					P_{trend}
	0	>0–0.5	>0.5–1	>1–2	>2	
**All-cause mortality**						
Excluding subjects with extreme values of energy intake[b]	1.00 (reference)	0.88 (0.83–0.93)	0.83 (0.78–0.89)	0.86 (0.80–0.92)	0.87 (0.81–0.92)	**0.011**
Excluding deaths observed within the first five years of follow-up	1.00 (reference)	0.88 (0.83–0.94)	0.83 (0.78–0.90)	0.86 (0.79–0.92)	0.88 (0.82–0.94)	**0.039**
Adjustment for propensity score on unadjusted model	1.00 (reference)	0.75 (0.71–0.79)	0.74 (0.70–0.79)	0.72 (0.67–0.77)	0.78 (0.73–0.82)	**0.005**
Additional adjustment for Healthy Eating Index-2015[c]	1.00 (reference)	0.89 (0.84–0.94)	0.84 (0.79–0.90)	0.86 (0.80–0.92)	0.86 (0.81–0.91)	**<0.001**
Additional adjustment for sodium, added sugars, and saturated fatty acids[d]	1.00 (reference)	0.88 (0.83–0.93)	0.83 (0.78–0.89)	0.85 (0.79–0.91)	0.84 (0.79–0.90)	**<0.001**
**Cardiovascular mortality**						
Excluding subjects with extreme values of energy intake[b]	1.00 (reference)	0.78 (0.70–0.86)	0.75 (0.66–0.84)	0.75 (0.66–0.86)	0.78 (0.69–0.87)	**0.027**
Excluding deaths observed within the first five years of follow-up	1.00 (reference)	0.82 (0.73–0.91)	0.78 (0.69–0.89)	0.78 (0.68–0.89)	0.82 (0.72–0.93)	0.099
Adjustment for propensity score on unadjusted model	1.00 (reference)	0.63 (0.57–0.69)	0.63 (0.56–0.70)	0.58 (0.51–0.65)	0.64 (0.57–0.71)	**<0.001**
Additional adjustment for Healthy Eating Index-2015[c]	1.00 (reference)	0.80 (0.72– 0.88)	0.76 (0.68–0.86)	0.76 (0.67–0.86)	0.77 (0.69–0.86)	**0.003**
Additional adjustment for sodium, added sugars, and saturated fatty acids[d]	1.00 (reference)	0.79 (0.71–0.87)	0.76 (0.67–0.85)	0.76 (0.67–0.86)	0.76 (0.68–0.85)	**0.003**
**Cancer mortality**						
Excluding subjects with extreme values of energy intake[b]	1.00 (reference)	0.95 (0.85–1.07)	0.94 (0.84–1.07)	0.98 (0.86–1.12)	0.99 (0.87–1.12)	0.470
Excluding deaths observed within the first five years of follow-up	1.00 (reference)	0.91 (0.81–1.03)	0.90 (0.79–1.03)	0.95 (0.83–1.10)	0.96 (0.84–1.09)	0.464
Adjustment for propensity score on unadjusted model	1.00 (reference)	0.87 (0.78–0.97)	0.91 (0.81–1.02)	0.91 (0.81–1.03)	0.95 (0.85–1.07)	0.075
Additional adjustment for Healthy Eating Index-2015[c]	1.00 (reference)	0.96 (0.86–1.07)	0.95 (0.84–1.07)	0.99 (0.87–1.12)	0.98 (0.87–1.10)	0.654
Additional adjustment for sodium, added sugars, and saturated fatty acids[d]	1.00 (reference)	0.95 (0.85–1.06)	0.94 (0.83–1.06)	0.98 (0.87–1.12)	0.97 (0.86–1.09)	0.701

[a]Values are hazard ratios (95% confidence intervals). Hazard ratios were adjusted for the following variables unless otherwise specified: age (years), sex (male, female), ethnicity (non-Hispanic white, non-Hispanic black, Hispanic, others), educational level (college below, college graduate, postgraduate), marital status (married or living as married, widowed, divorced, separated, never married), study center (10 categories), history of hypertension (yes, no), history of diabetes (yes, no), aspirin use (yes, no), hormone use status (current, former, never) for women, smoking status (current, former, never), alcohol consumption (g/day), body mass index (kg/m^2), physical activity (min/week), energy intake from diet (kcal/day), and consumption of red meat (g/day), processed meat (g/day), fruit (g/day), vegetable (g/day), whole grain (servings/day), dairy (cups/day), coffee (g/day), and tea (g/day). For all-cause and cancer mortality, the model was further adjusted for family history of cancer (yes, no).

[b]Extreme values of energy intake are defined as <800 or >4000 kcal/d for men and <500 or >3500 kcal/d for women.

[c]This covariate was treated as the continuous variable in multivariable Cox regression.

[d]These covariates were treated as the continuous variable in multivariable Cox regression.

Supplementary Table 2. Comparison of sociodemographic characteristics and medical histories between included and excluded populations[*].

Characteristics	Included population	Excluded population	Standardized difference
Number of participants	91891	62996	
Age (years)	65.3 ± 5.7	65.1 ± 5.8	0.033
Male	42543 (46.3)	29612 (47.0)	0.014
Ethnic group			
Non-Hispanic white	83548 (90.9)	56513 (89.7)	
Non-Hispanic black	2981 (3.2)	2120 (3.4)	0.063
Hispanic	1360 (1.5)	1458 (2.3)	
Others [†]	4002 (4.4)	2905 (4.6)	
Body mass index (kg/m^2)	27.2 ± 4.8	27.5 ± 5.1	0.070
Educational level			
College below	58056 (63.3)	38934 (67.3)	
College graduate	16299 (17.8)	9043 (15.6)	0.084
Postgraduate	17355 (18.9)	9872 (17.1)	
Smoking status			
Current	8473 (9.2)	5435 (9.4)	
Past	38347 (41.7)	24407 (42.0)	0.009
Never	45051 (49.0)	28223 (48.6)	
History of diabetes	5258 (5.8)	3865 (6.7)	0.039
History of hypertension	27745 (30.4)	17297 (30.0)	0.008
Family history of cancer	51525 (56.2)	31795 (55.0)	0.025

[*]Values are mean ± standard deviation or counts (percentage) as indicated.
[†]"Others" refers to Asian, Pacific Islander, or American Indian.

Supplementary Table 3. Distribution of variables with missing data before and after multiple imputation[a].

Variable	Before multiple imputation	After multiple imputation	Number (%) with missing data
Body mass index (kg/m^2)	27.1 ± 4.7	27.1 ± 4.8	1211 (1.32)
Physical activity (min/week)[b]	125.5 ± 122.5	122.6 ± 123.0	22617 (24.61)
Marital status			
Married	54007 (79.6)	71933 (78.3)	171 (0.19)
Widowed	5066 (7.5)	7405 (8.1)	
Divorced	6202 (9.1)	8915 (9.7)	
Separated	471 (0.7)	698 (0.8)	
Never married	2077 (3.1)	2940 (3.2)	
History of diabetes			
Yes	3317 (4.9)	5286 (5.8)	524 (0.57)
No	64506 (95.1)	86605 (94.2)	
History of hypertension			
Yes	19701 (29.0)	27915 (30.4)	508 (0.55)

No	48122 (71.0)	63976 (69.6)	
Educational level			
College below	41640 (61.4)	58165 (63.3)	181 (0.20)
College graduate	12547 (18.5)	16332 (17.8)	
Postgraduate	13636 (20.1)	17394 (18.9)	
Smoking status			
Current	5513 (8.1)	8478 (9.2%)	20 (0.02)
Former	28376 (41.8)	38353 (41.7%)	
Never	33934 (50.0)	45060 (49.0%)	

[a]Values are mean (standard deviation) or counts (percentage) as indicated.
[b]Total time of moderate to vigorous activity per week.
